# Small invasive colon cancer with systemic metastasis: A case report

**DOI:** 10.1186/1471-230X-11-59

**Published:** 2011-05-20

**Authors:** Minori Matsumoto, Takeshi Nakajima, Ken Kato, Tsutomu Kouno, Taku Sakamoto, Takahisa Matsuda, Ryoji Kushima, Yutaka Saito

**Affiliations:** 1 Endoscopy Division, National Cancer Center Hospital, 5-1-1 Tsukiji, Chuo-ku, Tokyo 104-0045, Japan; 2 Gastrointestinal Oncology Division, National Cancer Center Hospital, 5-1-1 Tsukiji, Chuo-ku, Tokyo 104-0045, Japan; 3 Breast and Medical Oncology Division, National Cancer Center Hospital, 5-1-1 Tsukiji, Chuo-ku, Tokyo 104-0045, Japan; 4 Pathology Division, National Cancer Center Hospital, 5-1-1 Tsukiji, Chuo-ku, Tokyo 104-0045, Japan; 5 Department of Medical Oncology, Sasaki Foundation Kyoundo Hospital, 1-8 Kanda Surugadai, Chiyoda-ku, Tokyo, 101-0062, Japan

**Keywords:** Nonpolypoid colorectal cancer, CDX2, CK20, CK7, systemic metastasis

## Abstract

**Background:**

Recently, especially in Japan, several researchers have suggested that colorectal cancer can develop not only through an adenoma-carcinoma sequence but also from normal mucosa via a *de novo *pathway, and that these *de novo *cancers have more aggressive malignant potential. We report a case of aggressive colon cancer resulting in systemic metastasis despite small tumour size.

**Case Presentation:**

A 35-year-old woman presented at the referring hospital with swelling of the left cervical lymph node. Biopsy of the lymph node revealed metastatic adenocarcinoma; however, CT scan and mammography were unable to identify the site of the primary lesion. She was diagnosed with unknown primary cancer and referred to our hospital for further examination. Immunohistochemical reevaluation showed the cervical lymph node biopsy specimen to be positive for CDX2 and CK20 and negative for CK7 expression, leading us to suspect the presence of a primary colorectal cancer. We performed a total colonoscopy, and detected a small protruding lesion in the transverse colon. The tumour was only 12 mm in diameter, with a central depressed component and a severely thickened stalk, which suggested direct cancer invasion of the deep submucosa. We concluded that this lesion was the site of origin of the metastasis despite the small tumour size, and performed diagnostic endoscopic mucosal resection. The lesion was found to have an intramucosal cancer component, demonstrating that this lesion represented primary colon cancer. The patient was referred to the gastrointestinal oncology division for systemic chemotherapy.

**Conclusions:**

In this case, immunohistochemical findings strongly suggested the existence of a colorectal cancer. The non-polypoid gross appearance of the tumour suggested that it can originate *de novo *, thus providing a valuable case in support of the aggressive malignant potential of a *de novo *colorectal cancer pathway.

## Background

Colorectal cancer is the second leading cause of cancer deaths in men and women in Western countries [1], and its incidence is gradually increasing in Japan as well. The most common pathway of colorectal cancer development is thought to be the adenoma-carcinoma sequence, in which carcinoma develops from an adenomatous polyp [2]. The current practice of removing adenomatous polyps of the colon and rectum is based on the belief that this will prevent colorectal cancer [3]. However, recent reports have described small depressed [4], leading to the proposal of an alternative pathway of *de novo *colon carcinogenesis, which involves an aggressive growth phenotype and quick infiltration of neighbouring tissue and lymph nodes [5-7]. The most common site of metastasis of these cancers is the liver, followed by the lung. Herein, we report a rare case of a small colon cancer with a depressed component and aggressive malignant potential with systemic metastasis, where the chief complaint was cervical lymph node swelling.

### Case Presentation

**Figure 1:**
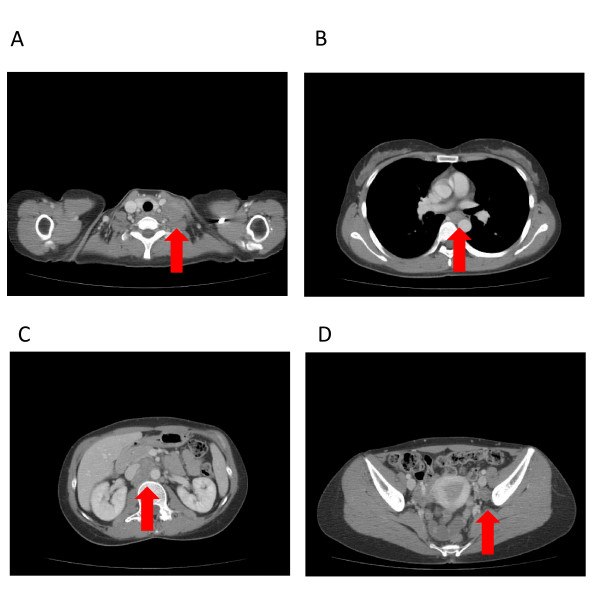
**Whole-body computed tomography (CT) findings**. Systemic lymph node swelling was observed. (A) Left supraclavicular lymph node metastasis measuring 33 mm in diameter (arrowhead). (B) Middle thoracic para-oesophageal lymph node metastasis measuring 24 mm in diameter (arrowhead). (C) Multiple para-aortic lymph node metastases in the abdomen, the largest one measuring 43 mm in diameter (arrowhead). (D) Left iliac internal lymph node metastasis measuring 18 mm.

In September 2009, a 35-year-old woman presented at the referring hospital with left cervical lymph node swelling. Malignant lymphoma (ML) was first suspected, but aspiration cytology and biopsy of the lymph node suggested metastatic adenocarcinoma resulting from breast cancer. However, no evidence of breast cancer was found on ultrasonography and mammography examinations. Whole-body computed tomography (CT) showed systemic lymph node swellings (left supraclavicular and multiple para-aortic lymph nodes) but no primary lesion (Figure 1). Upper Gastrointestinal endoscopy revealed no evidence of malignancy. The patient was diagnosed with unknown primary cancer and referred to our hospital for further examination in November 2009.

**Figure 2:**
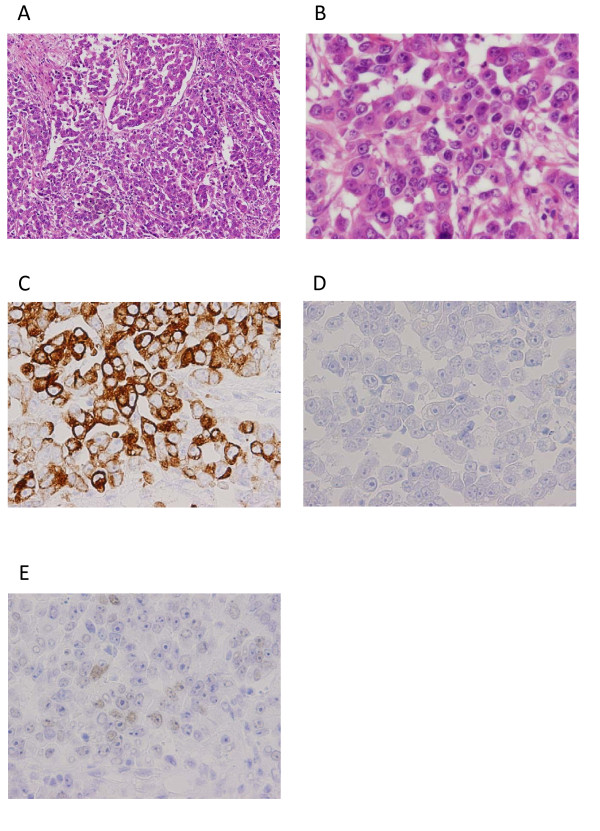
**Pathological findings of lymph node metastasis**. (A, B) Poorly differentiated adenocarcinoma was observed, with tumour cells spreading into fibrosis stroma in the tumour tissue. Tumour cells contained acidophilic vacuoles and oval nuclear bodies with several clear nucleoluses and partially formed glandular differentiations. (C) Immunohistochemical staining for CK20 was positive. (D) Immunohistochemical staining for CK7 was negative. (E) Immunohistochemical staining for CDX2 was weak but diffusely positive.

**Figure 3:**
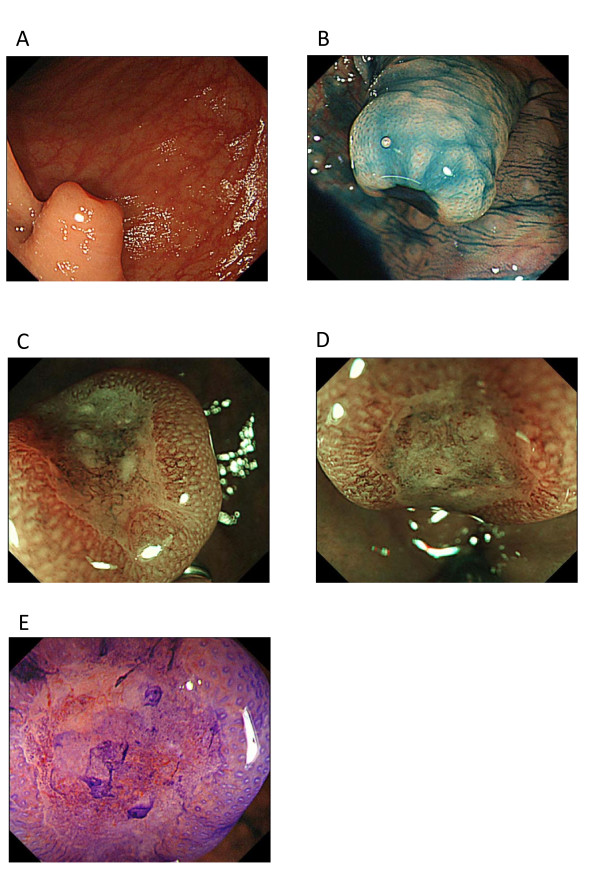
**Colonoscopy findings**. (A) Conventional colonoscopy showed a protruding lesion with central depression in the transverse colon, 12 mm in diameter. Its stalk was severely thickened, suggesting cancer invasion. (B) Chromoendoscopy with 0.4% indigo-carmine dye clearly showed the depressed area and non-neoplastic mucosa covering the edge of the cancer, suggesting that this tumour followed a non-polypoid growth (NPG)-type pattern. (C, D) Magnified NBI (Narrow Band Imaging) micrograph of the central depressed area, showing loose, irregular capillary vessels. We diagnosed the tumour as type IIIB according to Sano's classification, which suggests the possibility of deep cancer invasion of the submucosal layer. (E) Magnified view with 0.05% crystal violet staining of the surface of the central depression with a severe irregular pit pattern identified in the demarcated area.

We reevaluated the lymph node biopsy specimen obtained by the referring hospital. The tumour cells with oval to round nuclei with prominent nucleoli were found to be showing partial ductal structures, leading us to suspect poorly differentiated adenocarcinoma. Additionally, immunohistochemistry (IHC) studies showed the biopsy specimen to be negative for cytokeratin 7 (CK7), and positive for CDX2, an intestine-specific homeobox transcription factor, and cytokeratin 20 (CK20), a cytoskeletal protein usually found in the colonic epithelium. These IHC results strongly suggested the metastasis from a primary colorectal cancer (Figure 2). We performed a total colonoscopy and detected a small protruding lesion in the transverse colon (Figure 3). The tumour was very small, with a diameter of only about 12 mm, and contained a depressed component, leading to a diagnosis of macroscopic type with 0-Is+IIc according to the Paris classification [8]. Furthermore, the severely thickened stalk and irregular pits visible in a magnified image of the depressed area combined to suggest direct invasion of the deep submucosa (Figure 3C) [9]. We concluded that this lesion was the primary site of the metastatic cancer despite its small size.

**Figure 4:**
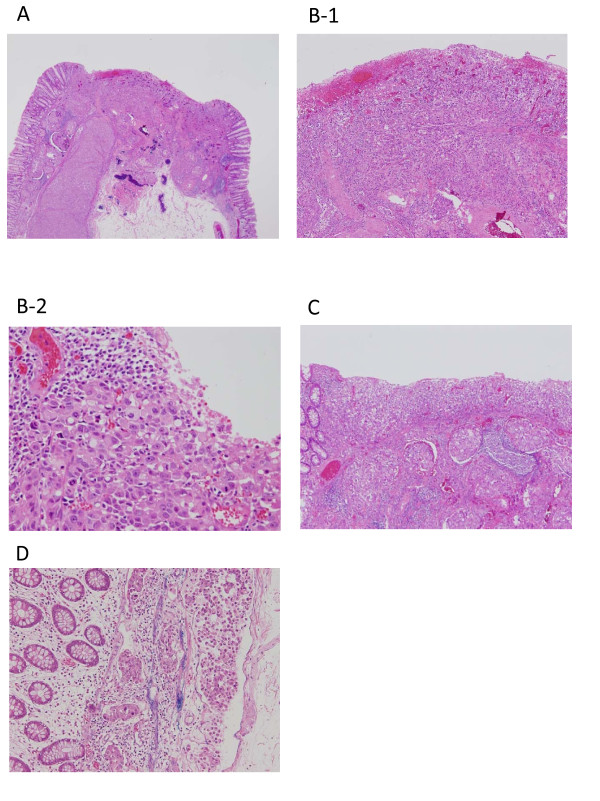
**Pathological results of endoscopic mucosal resection**. (A) An intramucosal component corresponding to the depressed lesion was observed. The tumour formed a massive invasion below the muscularis propria. (B-1,2) The tumour was composed of poorly differentiated adenocarcinoma and signet-ring cell carcinoma, mostly formed by solidly proliferating cells but with some indistinct gland formation. (C) The tumour had an intramucosal component, but infiltrated deeply into the submucosal layer with severe lymphatic and venous invasion. (D) Extensive lymphatic and venous invasion was detected mainly under the muscularis propria.

**Figure 5:**
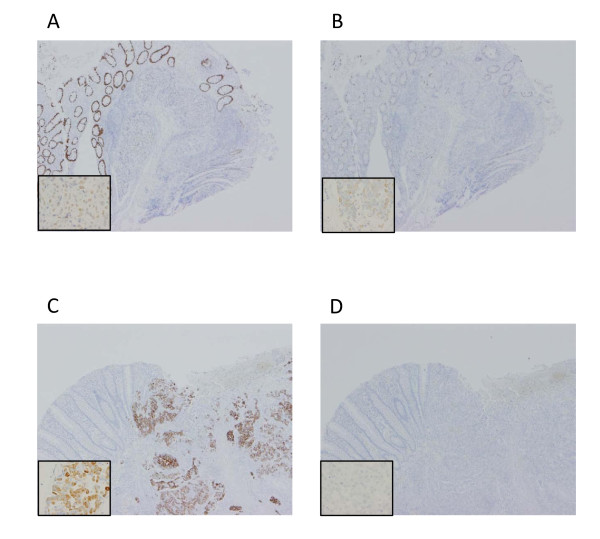
**Immunohistochemical investigation of the resected specimen**. (A) Weak CDX-2 expression was observed in the carcinoma. (B) MUC-2 expression was observed. (C) MUC5AC expression was observed. (D) MUC6 expression was not observed.

A diagnostic endoscopic mucosal resection (EMR) was performed and the specimen removed was serially dissected into three sections and examined. The tumour was composed of poorly differentiated adenocarcinoma and signet-ring cell carcinoma (Figure 4). It primarily consisted of solidly proliferated cells, but exhibited some indistinct gland formation. The tumour had a preserved intramucosal component but infiltrated deeply into the submucosal layer, with severe lymphatic and venous invasion detected the submucosal layer. The vertical and horizontal cut ends of the resected tumour were both positive for cancer cells (Figure 4). IHC studies showed strong expression of MUC5AC, and weak expression of MUC2 and CDX2; indicating the tumour having a mixed gastric and intestinal character, with the gastric phenotype being predominant. Patients with advanced colorectal cancer with gastric phenotypes have been reported to frequently exhibit lymphatic permeation and lymph node metastasis [10]. Additionally, the tumour was positive for CK20 and slightly positive for CK7. There was no evidence of differentiation to endocrine tumour or hepatoid adenocarcinoma (Figure 5).

These pathological results suggested that colon cancer was the origin of lymph node metastasis. The patient was referred to the Gastrointestinal Oncology Division, where she underwent combination chemotherapy using capecitabine (Xeloda^® ^) plus oxaliplatin (Elplat^® ^) and bevacizumab (Avastin^® ^), which is one of the standard therapy of metastatic colon cancer. After 4 courses, CT scan showed a significant reduction of tumour volume, but after 10 courses, the tumour marker rapidly increased and peritoneal metastasis progressed. The genomic analysis of KRAS status revealed wild type, so we started the second-line combination chemotherapy using cetuximab (Erbitax^® ^) plus irinotecan hydrochloride (Topotecin^® ^). After the 4 times administration of cetuximab, tumor marker was decreased significantly. Her age, i.e. 35-years, is in accordance with the Guideline, which are used as recommendation criteria for MSI testing. However, she did not agree to undergo MSI testing, because she would like to give top priority to receive the chemotherapy.

## Conclusions

In patients with an unknown primary carcinoma, the site remains unidentified in 15~25% of the cases even after autopsy [11,12], although recent advances in clinical examination and diagnostic work-up have decreased this frequency. The median survival of high-risk patients with cancer of unknown primary origin ranges from 3 to 11 months, making prompt diagnosis and treatment very important [13-15]. In this case, 2 months elapsed between the initial consultation at the referring institution and the patient's presentation at our hospital. Pathological and IHC reviews of cervical lymph node tissue were the main tools used to determine the primary site of cancer origin.

When attempting to trace the primary site of a metastatic tumour of unknown origin, clarification of the histological type is indispensable in selecting appropriate treatment. Microscopic features and cell morphologies can be identified with hematoxylin and eosin (HE) staining, and this information can be used to identify the primary site. However, in the evaluation of undifferentiated or poorly differentiated cancers, IHC evaluation is a useful addition to HE staining in locating the tumour site. In particular, a pattern of positive CDX2 or CK20 expression and negative CK7 expression is indicative of primary colorectal cancer [16,17].

In this case, IHC investigation of a lymph node biopsy strongly suggested primary colorectal cancer. In a total colonoscopy examination, we detected one colorectal lesion with the gross appearance of a submucosal tumour. This appearance is relatively rare in primary colon cancer, and thus we should exclude metastatic colon cancer as a possible diagnosis. EMR is more useful than simple biopsy in confirming the existence of a mucosal component, which would lead to a primary cancer diagnosis, but it carries a high risk of bleeding and perforation in cases where deep submucosal invasion is suspected. We performed diagnostic EMR after obtaining proper consent and agreement.

The adenoma-carcinoma sequence hypothesis proposes that colorectal cancers arise from adenomatous polyps. Alternately, several researchers, especially in Japan, have suggested that colorectal cancer can also develop from normal mucosa, in a *de novo *process involving morphological changes from a small superficial-type carcinoma to depressed-type carcinoma. *De novo *cancers are thought to have an aggressive growth phenotype, despite small tumour size, and to quickly infiltrate neighbouring tissue and lymph nodes. In this case, the tumour exhibited a severely thickened stalk, which suggested direct cancer invasion of the deep submucosa, and a clearly demarcated depressed area in the centre of the lesion, suggesting that it can originate *de novo *simply due to its morphology and clinical behaviour. This case of small primary colon cancer with systemic metastasis provides valuable support for the aggressive malignant potential of the *de novo *pathway in colorectal carcinogenesis.

In conclusion, we have reported a rare case of small primary colon cancer with systemic metastasis

## Abbreviations

CK: cytokeratin, CT: computed tomography, EMR: endoscopic mucosal resection, IHC: immunohistochemistry

## Competing interests

The authors declare that they have no competing interests.

## Authors' contributions

MM collected the data and wrote the report, and was involved in drafting the manuscript. TN revised the manuscript critically for important intellectual content. All authors read and approved of the final manuscript.

## Consent

Written informed consent was obtained from the patient for publication of this case report and any accompanying images. A copy of the written consent is available for review by the Editor-in-Chief of this journal

## Pre-publication history

The pre-publication history for this paper can be accessed here:

http://www.biomedcentral.com/1471-230X/11/59/prepub
